# Supplementary material for: The Real-World Evidence on the Fragility and Its Impact on the Choice of Treatment Regimen in Newly Diagnosed Patients with Multiple Myeloma over 75 Years of Age
Source: Cancers (Basel). 2023 Jul 2;15(13):3469. doi: 10.3390/cancers15133469 (PMC10340664; doi:10.3390/cancers15133469)
Supplement: Supplementary file 1 [file cancers-15-03469-s001.zip › cancers-2450326-supplementary.pdf]

Supplementary Materials

Table S1. Fragility subgroup analysis for the FIRST study. Adapted from [14, 15].

**Median PFS (months)**	**Fit patients (*fit*)**	**Intermediately fit (*intermediate patient*)**	**Frail patients (*frail*)**
Median PFS (months) for Rd	43.7	31.1	20.3
Median PFS (months) for MPT	23.9	22.6	20.2
Hazard ratio	0.56; 95% CI (0.38-0.84)	0.62; 95% CI (0.46-0.85)	0.79; 95% CI (0.64-0.97)

Abbreviations: CI – confidence intervals; MPT – melphalan-prednisone-thalidomide; PFS – progression-free survival; Rd - lenalidomide-dexamethasone.

Table S2. Fragility scale according to the International Myeloma Working Group.

**Age (in years)**	**Result**
≤75	0
75–80	1
>80	2
**Katz Daily Fitness Scale**	
>4	0
≤4	1
**Lawton's Advanced Activities of Daily Living Scale**	
>5	0
≤5	1
**Charlson Comorbidity Scale**	
≤1	0
≥2	1
**RESULTS**	
Fit/ Efficient	0
Intermediately fit	1
Fraily/ Fragile	≥2

Table S3. International Myeloma Working Group scale for patients over 75.

Intermediately fit	Fraily
Age 76-80 or ADL ≤ 4 or I-ADL ≤ 5 or CCI ≥ 2	1. Age > 80 regardless of ADL, I-ADL, CCI<br>2. 76–80 years old and both ADL ≤ 4, I-ADL ≤ 5, CCI ≥ 2.<br>3. Age ≤ 75 years old and at least 2 with ADL ≤4, I-ADL ≤5, CCI ≥2.
Recommended treatment: reduction of treatment intensity.	Absolute dose reduction.
Reduced three-drug or full/reduced two-drug regimens.	Two-drug-reduced regimens.<br>The most important – palliative and supportive treatment.

Abbreviations: ADL – Katz Scale; CCI – Charlson Comorbidity Index; I-ADL – Lawton Scale.

Table S4. Comparison of fitness scales for the elderly with multiple myeloma.

Scale	Biological factors	Performance test	Comparison with IMWG	Origin of the study population	Prospective assessment
IMWG (Palumbo)	-age<br>-CC*	-Katz scale<br>-I-ADL scale	not applicable	from clinical trials	No
R-MCI	-age<br>-lung diseases<br>-kidney disease (eGFR)	-performance status according to Karnofsky<br>-assessment of functioning: subjective assessment or geriatric "Get up and go" test or I-ADL	Yes	-from clinical trials<br><br>-general population	No
Mayo Risk Score (MRS)	-age<br>-NT-proBNP	-ECOG performance status (WHO)	No	-from clinical trials<br>-general population	No

* CCI (Charlson-comorbidity-index)

* Revised Myeloma Comorbidity Index

Abbreviations: ECOG - Eastern Cooperative Oncology Group; eGFR – estimated glomerular filtration rate; I-ADL – Lawton Scale; IMWG – International Myeloma Working group; NT-proBNP - N-terminal pro-brain natriuretic peptide; WHO –World Health Organization.

Table S5. Response to first line treatment depending on whether drugs were modified or not.

Response for first Line Therapy	Treatment with schema **without** modification drugs	Treatment with schema **with** modification drugs	p-val
Complete Response (CR)	12 (8.4%)	6 (11.1%)	0.084
Very good Partial Response and Partial Response (VGPR and PR)	98 (68.5%)	27 (50.0%)	
Stable Disease (SD)	21 (14.7%)	15 (27.8%)	
Progressive Disease (PD)	12 (8.4%)	6 (11.1%)	

Table S6. Use of regimens with or without drug modification.

Schema in first line therapy	Modification		p- val
	Without modification drugs	With modification	
3 –drug schema	122 (85.3%)	35 (64.8%)	0.003
2 – drug schema	21 (14.7%)	19 (35.2%)	

Table S7. Use of regimens with or without drug modification according of frailty.

Fragility scale (International Myeloma Working Group)	First line therapy			
	Treatment with 3 drug regimen		Treatment with 2 drug regimen	
	Without modification drugs (%)	With Modification drug	Without modification drugs	With Modification drug
**Intermediately fit**	27 (62.8%)	7 (27.2%)	5 (55.6%)	4 (44.4%)
	34 (85.0%)		9 (15.0%)	
**Frailty**	95 (77.2%)	28 (22.8%)	16 (51.6%)	15 (49.4%)

	123 (79.8%)		31 (20.8%)	
**Total number**	122 (77.7%)	35 (22.3%)	21 (52.5%)	19 (47.5%)
	157 (79.7%)		40 (20.3%)	
Frailty (only 80 years old & older)	35 (70.0%)	15 (30.0%)	6 (40.0%)	9 (60.0%)
	50 (76.9%)		15 (23.1%)	

Table S8. The relationship between the number of comorbidities identified in each patient and the treatment protocol selected.

**Number of comorbidities**	**Number of cases (%)**	**Number (%) of patients treated with a 2-drug vs. a 3-drug regimen**		**Trend evaluation p-value**	**Independence assessment p-value**
		**3-drug schema**	**2-drug schema**		
None	21 (11.0%)	18 (85.7%)	3 (14.3%)		
1	35 (18.0%)	27 (77.1%)	8 (22.9%)		
2	54 (27.0%)	44 (81.5%)	10 (18.5%)	0.335	0.395
3	34 (17.1%)	30 (88.2%)	4 (11.8%)		
4 or more	53 (26.9%)	38 (71.7%)	15 (28.3%)		

Table S9. Responses to bortezomib use in first-line treatment.

**I line**		**Response to first-line treatment**				
		**Complete remission (CR)**	**Very good partial response (VGPR)**	**Partial response (PR)**	**Disease stabilization (SD)**	**Disease progression**
**bortezomib**	Not used	11 (9.6%)	6 (5.3%)	71 (62.3%)	18 (15.8%)	8 (7.0%)
	Used	7 (8.4%)	9 (10.8%)	39 (47.0%)	18 (21.7%)	10 (12.0%)

Table S10. The relationship between co-existing disease and the treatment protocol selected.

	Number of cases/N important* cases/total*	Number of patients with a given treatment regimen (%)		Independence assessment p-value
		**3-drug**	**2-drug**	
Total	197/197	**157 (100%)**	**40 (100%)**	
Presence of cardiovascular disease (any)	166/197	129 (82.2%)/157	37 (92.5%)/40	0.145
Hypertension	158/197	124 (79.0%)/157	34 (85.0%)/40	0.507
Ischemic heart disease	58/197	46 (29.3%)/157	12 (30.0%)/40	1
Circulatory failure	50/161	38 (30.9%)/123	12 (31.6%)/38	1
Kidney disease	48/133	33 (32.7%)/101	15 (46.9%)/32	0.204*
Respiratory disease	40/197	34 (21.7%)/157	6 (15.0%)/40	0.509
Diabetes	40/197	32 (20.4%)/157	8 (20.0%)/40	1
Gastritis/GERD	29/138	21 (19.8%)/106	8 (25.0%)/32	0.621
Other cancer types	28/145	22 (19.5%)/113	6 (18.8%)/32	1
Liver dysfunction	21/197	16 (10.2%)/157	5 (12.5%)/40	0.774
Cerebral circulation disorders	18/197	13 (12.3%)/106	5 (16.1%)/31	0.556

** missing data have been omitted*

Second-Line Treatment

Within 12 months of diagnosis, 84 (42.6%) individuals received second-line treatment, of which 28 (33.3%) were due to disease resistance to first-line of treatment, and 56 (66.7%) were due to progression after the previous response (response to treatment lasted more than 60 days) (Tables S6 and S9).

During the second line of treatment, three-drug and two-drug regimens were used (Table 9). Two-component schemes were chosen more often in the second line and were

used in 54 patients (64.3%), with the remaining 30 (35.7%) receiving three-component schemes. Among those who received a three-component first-line treatment, 44 (66.7%) received a two-component second-line treatment, and 22 (33.3%) received another three-component treatment. Ten (55.6%) of the patients who received a first-line two-drug treatment also received two-drug second-line treatment, while the other eight patients (44.4%) received a three-drug treatment (Table S9).

The reimbursement possibilities in Poland probably dictated the frequency of choosing a two-component treatment in the second line of treatment. Therefore, it is difficult to determine the dependence of such a choice. However, it should be noted that a three-drug regimen was used more often in patients after a previous two-drug regimen (44.4% vs. 33.3% for patients previously treated with a three-drug regimen).

Table S11. The second-line treatment used.

	**First-line treatment**	**Number of patients (n)**	**Number of patients (%)**	**Second-line treatment**	**Number of patients (n)**	**Number of patients (%)**
**First line of treatment**	3-component scheme	66	78.6%	3-component scheme	22	33.3%
				2-component scheme	44	66.7%
	2-component scheme	18	21.4%	3-component scheme	8	44.4%
				2-component scheme	10	55.6%

Table S12. Analysis of deaths.

	Cause of death	Cases (% of patients, N=197)	The treatment respectively: in the first line and in the second line		ECOG		ADL (according to Katz)			Number of comorbidities	
			3-drug schema	2-drug schema	0–2	3–4	Fully independent	Moderate impairment	Completely dependent	Below 4 comorbidities	4 or more comorbidities
Deaths in the first line of treatment	Disease progression	6 (3.0)	5	1	2	4	2	2	2	4	2
	Infection	5 (2.5)	4	1	5	0	5	0	0	2	3
	Other reason	3 (1.5)	3	0	3	0	3	0	0	1	2
Deaths in the second line of treatment	Disease progression	9 (4.6)	4	5	8	1	8	no data	no data	6	3
	Infection	2 (1.0)	2	0	1	1	1	0	1	1	1
	Other reason	0 (0.0)	0	0	0	0	0	0	0	0	0
Total number of deaths	Disease progression	15 (7.6)	9	6	10	5	13	0	0	10	5
	Infection	7 (3.6)	6	1	6	1	6	0	1	3	4
	Other reason	3 (1.5)	3	0	3	0	3	0	0	1	2
	**Total number of deaths (% of deaths)**	**25 (100)**	**18 (72.0)**	**7 (18.0)**	**19 (76.0)**	**6 (24.0)**	**19 (76.0)**	**2 (8.0)**	**3 (12.0)**	**14 (56.0)**	**11 (44.0)**
